# Guided growth in the correction of knee deformity in patients with congenital insensitivity to pain

**DOI:** 10.1186/s13018-021-02304-w

**Published:** 2021-03-11

**Authors:** Soroush Baghdadi, Sadegh Saberi, Taghi Baghdadi

**Affiliations:** Joint Reconstruction Research Center, Department of Orthopedics, Tehran University of Medical Sciences, Keshavarz Boulevard, Tehran, 1419733141 Iran

**Keywords:** Congenital insensitivity to pain, Hereditary sensory and motor neuropathy, Guided growth, Knee deformity, Hemiepiphysiodesis, Genu valgum

## Abstract

**Background:**

Orthopedic manifestations of congenital insensitivity to pain (CIP) can be devastating if left untreated. Knee deformities are common in patients with CIP and might lead to joint destruction and loss of walking ability. The purpose of the present study was to report the results and complications of guided growth procedures around the knee in patients with CIP.

**Methods:**

In a retrospective review, all patients with CIP who underwent guided growth procedures around the knee from 2009 to 2017 at a tertiary referral hospital were evaluated. Patients with secondary insensitivity to pain (e.g., syringomyelia), as well as patients with incomplete records, were excluded. Demographic data, clinical findings, correction rate, and complications were recorded.

**Results:**

Ten knees in six patients fulfilled the inclusion criteria. The median age was 10 (range, 5–12), with a mean follow-up of 31 months (range, 16–56). Distal femoral tension-band hemiepiphysiodesis was the most common procedure, followed by proximal tibial hemiepiphysiodesis. The mean correction rate was 0.28°/month for femoral deformity. Staples were removed prematurely in one patient due to extrusion. No cases of infection or skin dehiscence were observed. None of the patients needed a reconstructive knee procedure during the study period.

**Conclusions:**

The findings of this study suggest that guided growth procedures might have a role in the correction of knee deformities in patients with CIP. However, the correction rate is lower than that of typically developing children, patients should be closely followed to prevent complications, and stringent patient selection criteria should be followed to ensure success.

## Background

Congenital insensitivity to pain (CIP) is an umbrella term for a group of extremely rare disorders characterized by the congenital absence of nociception. Affected individuals are unable to perceive physical pain [1]. Dearborn was the first to describe the disorder with the term “congenital pure analgesia” [2]. However, as more cases were identified with heterogenous clinical presentations, attempts were made to classify disorders with CIP. Currently, CIP disorders are classified under hereditary sensory and autonomic neuropathies (HSAN). The most commonly accepted classification of HSANs was introduced by Dyck and Ohta in 1975 [3], with four types based on the phenotypic characteristics of patients. This classification has been expanded to encompass a variety of genetic disorders, which entail a lack of nociception as a common symptom [4]. The genetic underpinnings of HSAN have been extensively studied, with multiple genes and inheritance patterns found responsible for the disease spectrum, which ranges from pure absence of nociception to severe intellectual disability with self-mutilating behavior and early death [5, 6].

The lack of pain perception leads to the classic findings of painless venipunctures and injection from infancy; multiple painless fractures and dislocations, burns, cuts, and bruises; and loss of teeth and tongue injuries [1]. The earlier reports emphasized the limited life span of affected individuals, especially in male patients [7–9]. However, with increasing awareness of the disease on the one hand, and expansion of the definition of CIP to include less severe cases and related entities on the other, there seems to be a change in the age range of patients in the literature, with patients even being diagnosed in their 50s and 60s [10, 11].

Although surgical procedures are not unprecedented in individuals with CIP, the general consensus is that joint deformities and dislocations will have a poor prognosis despite any surgical intervention [9]. Reconstructive hip procedures have especially been unsuccessful, with reports of patients dislocating their hips after surgery and losing walking ability [8, 12].

With the growing life expectancy of patients with CIP thanks to earlier diagnosis, implementing injury prevention measures, and earlier treatment of life-threatening injuries, we felt that prevention of an unreconstructible knee deformity might be possible in certain individuals with CIP. Therefore, we have performed guided growth procedures for certain CIP patients in the last 10 years. We hypothesized that by performing a relatively simple procedure in skeletally immature patients, later deformities and dislocations, which can be devastating around the knee, could be prevented. This study aims to report the results and complications of guided growth procedures performed on a series of patients with CIP.

## Methods

In an IRB-approved retrospective chart review, records were searched for patients diagnosed with “congenital insensitivity to pain” who underwent guided growth procedures around the knee, including hemiepiphysiodesis with plates or staples from January 2009 (the year our electronic database was implemented) to December 2017. All surgeries were performed by the senior surgeon (TB) at a tertiary referral hospital. Patients were included if complete pre- and postoperative data were available for at least 1 year after surgery. Also, charts were thoroughly inspected to exclude patients with similar diagnoses, which might be misinterpreted as CIP (e.g., syringomyelia).

Demographic characteristics, presenting symptoms and other clinical findings, orthopedic manifestations, phenotypic classification (according to Dyck [3]), and genetic studies (when available), as well as the results and complications of the guided growth procedures, were recorded. Pre- and postoperative radiographs were obtained, along with clinical photographs, when available. Standing long-leg radiographs were reviewed, and the mechanical axis of the lower limb, mechanical lateral distal femoral angle (mLDFA), and mechanical medial tibial angle (mMPTA) were measured on pre- and postoperative radiographs to determine the correction rate.

The surgical technique was standard and has previously been described [13, 14]. The decision to offer a guided growth procedure was not standardized and was decided on a case-by-case basis. However, younger patients with no to mild intellectual disability who have a mild knee deformity and no subluxation are generally better candidates for guided growth. We also do not offer elective surgeries to CIP patients with severe intellectual disability, autoamputations, and self-mutilating behavior (hands or feet). Although the latter criterion was chosen arbitrarily, we believe that the presence of such phenotypes suggests a more severe disease and might be indicative of a poor prognosis.

Data collection and analysis were conducted using Microsoft Excel for Windows (Microsoft, Redmond, WA). Descriptive statistics were used to report the results.

Parents/legal guardians of all patients signed a written consent form. Patients were also briefed on the procedure and verbally consented to the procedure. Also, informed consent for publication of identifying information/images in an online open-access publication was obtained for patients whose images are published.

## Results

In total, ten knees in six patients fulfilled our inclusion criteria, of which four (67%) were male. The median age at surgery was 10 years (range, 5–12). Patients were followed for a mean of 31 months (range, 16–56).

Five patients were already being treated at a multidisciplinary clinic and were diagnosed with CIP prior to the referral for a knee deformity. One patient (case 5 in Table 1) was diagnosed with CIP after presenting to the pediatric orthopedics clinic with unilateral knee valgum deformity. Of note, her mother insisted that the patient had a painful knee, although the child had no pain on physical examination, and a genetic mutation was later found.

**Table 1:** Patient characteristics, outcomes, and complications in this series. ADHD = attention-deficit/hyperactivity disorder, ID = intellectual disability, HE = hemiepiphysiodesis, TBP = tension-band plating, SSR = sympathetic skin reflex, f/u = follow-up

Case no.	Age at surgery/ gender	Presenting symptoms	Other findings	Phenotypic classification (Dyck [3])	Knee deformity	Procedure	Outcome	Complications	Comments
1	11/M	Bilateral knee swelling	No anhidrosis, normal intelligence	V	Bilateral valga Left mLDFA = 73° Right mLDFA = 75°	Bilateral femoral HE with staples	Left mLDFA = 76° Right mLDFA = 80°	Staple extrusion necessitating removal	The patient is ambulatory with braces, right hip dislocation
2	9/M	Bilateral knee deformity, suspected infection	Anhidrosis, lip bites, tooth loss, dystrophic nails, ADHD, previous femoral fracture (Fig. 3)	IV	Bilateral valga Left mLDFA = 69° Right mLDFA = 72°	Bilateral femoral HE with TBP	Left mLDFA = 77° Right mLDFA = 80°	Post-op agitation	Absent SSR was found on neurophysiologic studies
3	12/M	Bilateral knee deformity	Anhidrosis, lip and tongue bites, moderate ID, ADHD, dystrophic nails	IV	Bilateral valga Left mLDFA = 77° Right mLDFA = 76°	Bilateral femoral HE with TBP	Left mLDFA = 82° Right mLDFA = 81°	None	The patient died of injuries following a fight at age 16
4	11/F	Windswept knee deformity	No anhidrosis, previous femoral fracture, normal intelligence, anosmia	V	Left genu varum (MPTA = 79°), right valgum (mLDFA = 79°)	Left tibial and right femoral HE with TBP	left MPTA = 83° right mLDFA = 85°	None	Lost to f/u after 2.5 years
5	5/F	Left knee swelling and deformity.	Anhidrosis, normal intelligence, bilateral ankle swelling (Fig. 1)	V	Left genu valgum mLDFA = 76°	Left femoral TBP with imbrication of medial structures	mLDFA = 84°	None	The patient had a proximal femoral fracture 1.5 years after the index surgery
6	12/M	Left tibial fracture at age 1, deformity of the same limb	Anhidrosis, bilateral ankle and foot swelling, metatarsal fracture, dystrophic nails (Fig. 2)	IV	Left genu valgum mLDFA = 75°	Left femoral HE	mLDFA = 84°	None	Tibial fracture had abundant callus and was biopsied

According to Dyck’s classification, three patients were grouped as type IV and three as type V. Genetic studies were offered to all patients, but one patient refused on financial grounds. Among the five patients for whom genetic studies were done, three were found to have a genetic mutation, but no mutations were identified in two patients. It should be noted that comprehensive genomic sequencing is not routinely performed at our institution due to high costs, and instead, single-gene testing is done based on the phenotypic presentation of the patient.

**Fig. 1:**
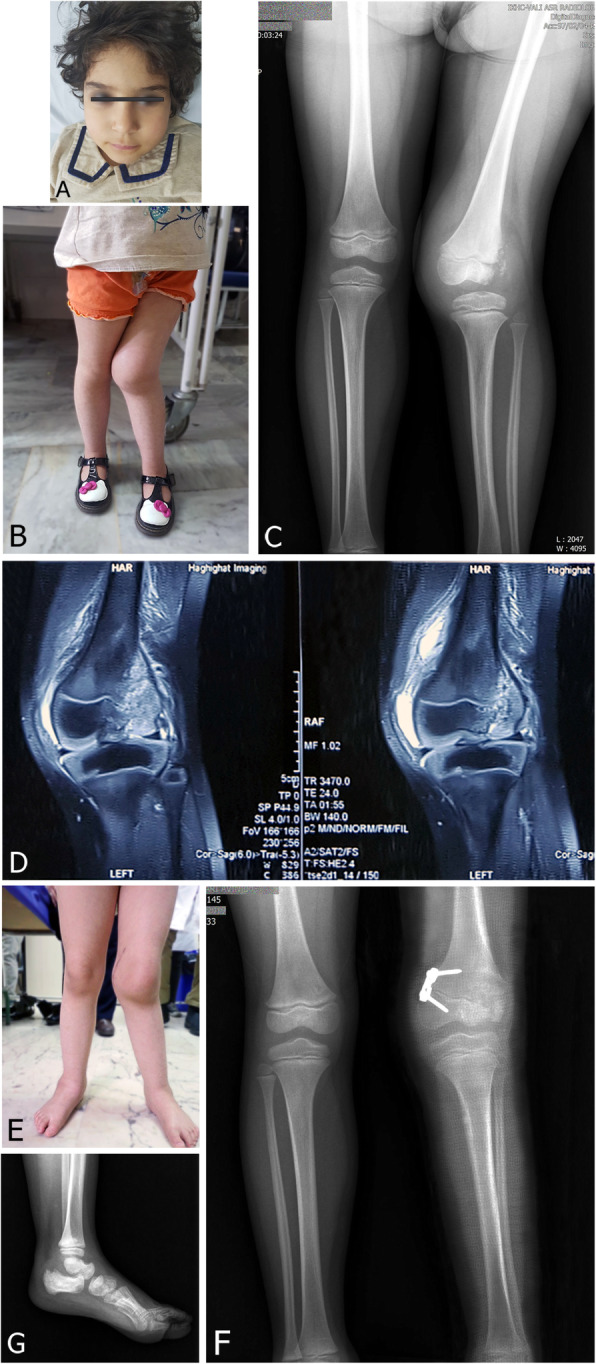
A five-year-old female (case 5) with HSAN type V. **a** No facial stigmata are present. **b** Clinical photograph at presentation, with massive knee effusion, left genu valgum, and difficulty walking. **c** Standing anteroposterior radiograph at presentation shows bone resorption in the lateral femoral condyle, increased joint space medially, and soft-tissue shadow indicative of knee effusion. **d** T2-weighted fat-saturated coronal magnetic resonance (MR) images show a lesion in the lateral femoral condyle, which suggests that the primary injury might have been a displaced Salter-Harris type IV lateral femoral condyle fracture. **e** Clinical photograph, 16 months postoperative. Left knee alignment has improved. Note that the incision is healed completely, and also the right ankle is swollen. **f** Standing AP radiograph at the same visit, showing an improved alignment of the left knee, consolidation of the initial lateral bone lucency, and improved joint congruency. The patient uses elastic bandages to decrease the swelling. **g** Lateral radiograph of the right ankle. Soft-tissue swelling and a sclerotic calcaneus are visualized, which indicate a probable bony injury. Short-term bracing was done for her ankle

**Fig. 2:**
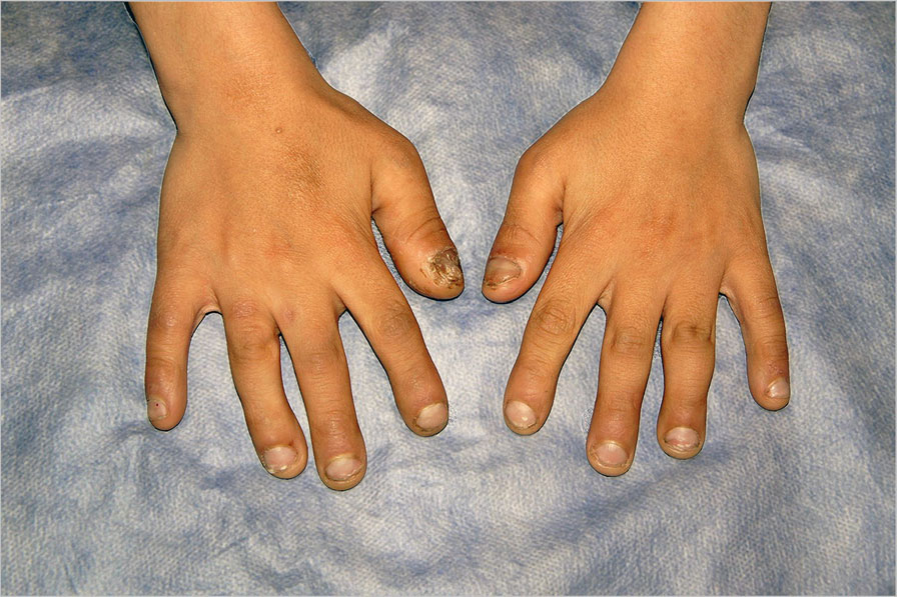
Clinical photograph of the hand of case 6, a 12-year-old male. Despite being classified as HSAN type IV and dystrophic nails, he has normal intelligence and no self-mutilating behavior, the presence of which we require to offer surgery to patients

**Fig. 3:**
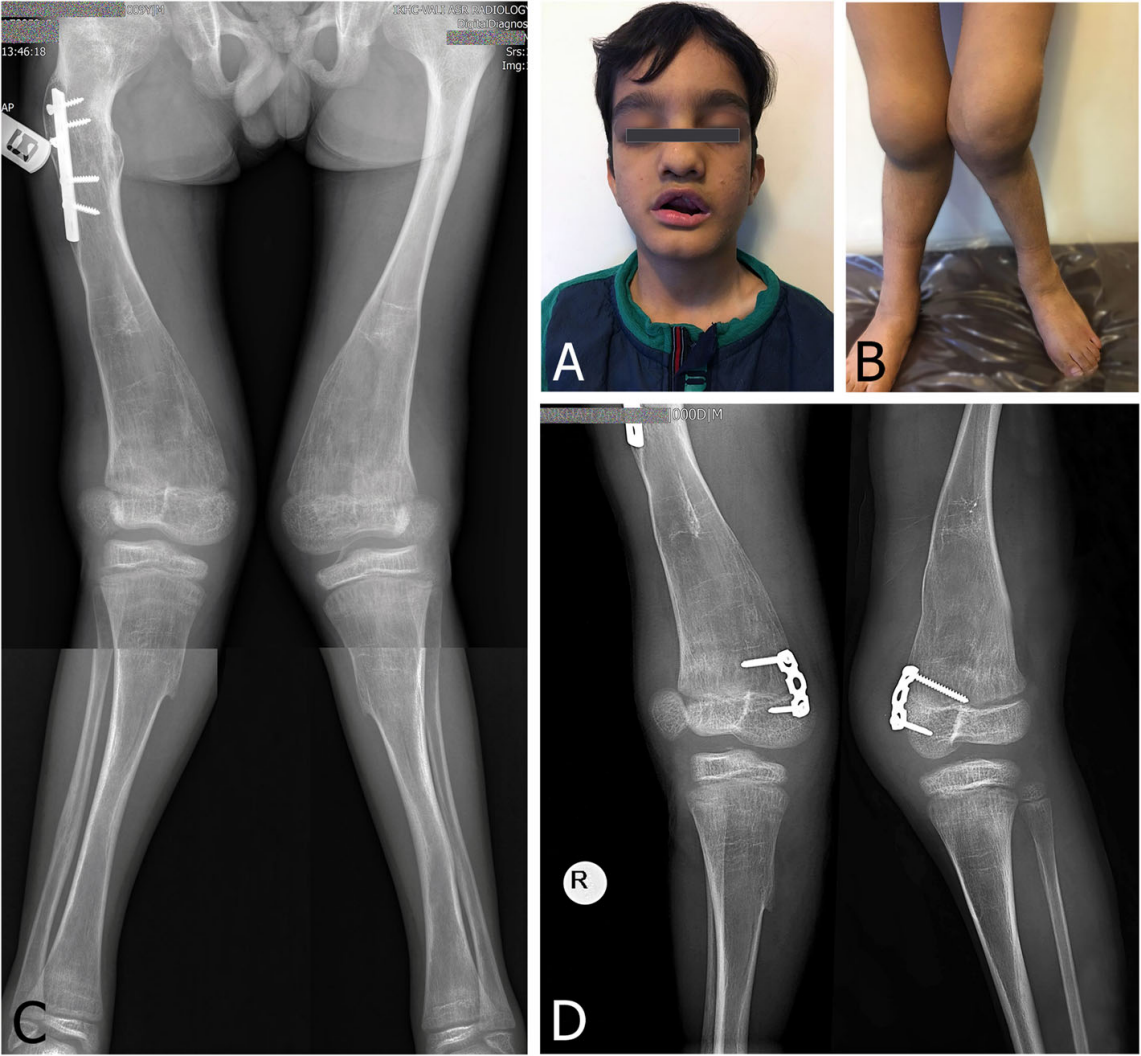
**a** Case 2, a 9-year-old male with HSAN type IV lip and tongue bites and loss of multiple teeth. However, this patient did not show self-mutilating behavior. **b** Clinical photograph at presentation, with severe bilateral swelling of the knees, and genu valga. **c** Standing AP radiograph, showing genu valgum. A previous femoral fracture was fixed with a plate. **d** Standing anteroposterior radiograph 2 years postoperative, showing improved lower limb alignment. The patellae seem dislocated on both radiographs. However, this patient has severe rotational malalignment of the lower limbs, with “apparent” patellar dislocation due to tangential X-ray projection

The phenotypic presentation of CIP was heterogeneous and ranged from no skin stigmata (case 5, Fig. 1a) to dystrophic nails (case 6, Fig. 2) and to lip and tongue bites and tooth loss (case 2, Fig. 3a).

Orthopedic manifestations included previous femoral fractures in two children, both treated with open reduction and internal fixation. Also, one patient (case 6) was diagnosed with CIP after a tibial fracture at age 1. This patient was first suspected of being a victim of child abuse, and the tibia was later biopsied because of hypertrophic callus formation. However, the biopsy showed normal regenerate bone and the fracture united uneventfully.

Bilateral genu valgum was the most common deformity and was present in three patients. Unilateral valgum was seen in two patients, and one patient had a windswept deformity, with the left knee in varus and the right knee in valgum. Distal femoral tension-band plating (TBP) was the most common procedure (6/10 knees), followed by distal femoral staple hemiepiphysiodesis (2/10 knees). One patient was treated for tibial varus with lateral proximal tibial TBP, and one case of permanent distal femoral hemiepiphysiodesis with a screw was also performed. The only anesthesia-related complication occurred in case 2, who was extremely agitated in the post-anesthesia care unit and was managed with sedatives.

The mean preoperative mechanical lateral distal femoral angle (mLDFA) in the nine knees with a distal femoral deformity was 74.6° (range, 69–79°), with a mean final mLDFA of 81° (range, 76–84°) and a mean correction rate of 0.28°/month. In the knee with a proximal tibial deformity, a preoperative medial proximal tibial angle (MPTA) of 79° was corrected to 83° at the final follow-up. Although full radiographic correction was achieved in only two knees, some degree of correction was observed in all knees. Only one procedure-related complication occurred during the study period, which was unilateral extrusion of staples in case 1. Staples were removed bilaterally to prevent asymmetric growth. This patient is still able to ambulate, despite the genu valga and right-sided hip dislocation. Figures 1a–g and 3a–g provide two examples of preoperative findings and outcome of treatment.

One patient (case 3) died 4 years after surgery after being involved in a street fight. This patient had behavioral issues and intellectual disability and had dropped out of school 2 years prior to the incident.

One patient (case 5) had a proximal femoral fracture 1.5 years after the index surgery, which was treated by surgical fixation and has healed at the latest follow-up.

No cases of infection, skin dehiscence or other procedure-related complications were observed during the study period.

## Discussion

In the present study, data from patients with congenital insensitivity to pain who underwent guided growth procedures around the knee were reviewed. Our results suggest that in carefully selected CIP patients, correction of the knee deformity might be achieved with a low rate of complications. Our primary goal in offering elective surgery to CIP patients is the prevention of devastating complications (including knee subluxation and dislocation) and not cosmesis. None of our patients experienced worsening of the knee deformity or subluxation/dislocation. However, this study is limited by its design and rarity of the disease and cannot establish a causal relationship/

CIP is extremely rare, with the largest populations of patients being reported from Israel [15] and Japan [16]. The genotypic and phenotypic characteristics of patients are heterogeneous, and multiple genetic mutations and phenotypes are reported in the literature [6, 17–19]. Dyck’s classification is used by most clinicians, and although it might not include every possible disease manifestation, being based on phenotypic characteristics makes this classification relevant in the clinical setting. Type I (which has at least 6 subtypes) is the most genetically heterogeneous and is believed to be the most common form of CIP [20]. It is the only HSAN showing autosomal dominant inheritance pattern and is generally diagnosed in early adulthood [10]. Therefore, unless diagnosed early, these patients would not be candidates for guided growth procedures.

Type II is characterized by early-onset sensory deficit, anhidrosis, autonomic dysfunction including recalcitrant constipation, recurrent ulcers and paronychia of the fingers and toes, and apnea, in addition to self-mutilation [6]. Severe medical issues preclude any elective surgical procedure in these individuals, and we did not have any patients with HSAN type II in this series.

Type III, also called familial dysautonomia or Riley-Day syndrome, is most commonly diagnosed based on sympathetic autonomic dysfunction, which could manifest as nausea and vomiting, tachycardia, hypotonia, orthostatic hypotension, and decreased or absent lacrimation [6]. Intellectual disability, along with the increased risk of anesthesia-related complications, renders this group of patients poor surgical candidates. HSAN type III is essentially limited to children of Ashkenazi Jewish descent, and we have not diagnosed any at our institution [21].

Type IV, also known as congenital insensitivity to pain with anhidrosis (CIPA) is an autosomal recessive disorder, diagnosed in childhood, and is characterized by anhidrosis, varying degrees of intellectual disability, and loss of oral sensation, which leads to face and mouth mutilation (Fig. 3a) [6]. Three of our patients (five knees) were categorized in this group. Interestingly, all suffered from lip and tongue bites and loss of teeth, but with no finger or toe loss (Fig. 2), which suggests different mechanisms for these two seemingly similar manifestations. All three also had dystrophic nails.

Patients with HSAN type V generally have no intellectual disability, although joint deformities are common [22]. Three of our patients were in this group. All had normal facies (Fig. 1a) and intelligence.

While we did not select patients based on their phenotypic classification, we only had type IV and V patients in this study. However, other types of HSAN are generally not ideal candidates for guided growth, due to the typical age at diagnosis (type I) and severe intellectual disability and self-mutilating behavior (type II), and intellectual disability with comorbid neurologic dysfunction (type III). Additionally, nine deformities in this series were valgus, and only one case of tibia vara. While the literature is not conclusive, a valgus deformity is to be expected in CIP, as patients are typically diagnosed during the growth period where a physiologic genu valgum is present. The repeated microinjuries due to the lack of pain and proprioception concentrates the abnormal forces on the lateral hemijoint and leads to a progressive valgus deformity, instability and, if left untreated, dislocation.

There are no reports of elective surgeries performed to prevent secondary complications in patients with CIP, but the results of corrective procedures have generally not been promising. Specifically, hip subluxations and dislocations do poorly after reconstructive surgery [12]. We believe that just as patients with no underlying neuropathy tolerate hip dislocation for decades with minimal limitations, the ambulatory status of CIP patients is not dependent on reduced hip joints, and reconstructive hip surgeries should not be offered to these individuals. However, at least one report of total hip arthroplasty has been published, with no short-term complications [23].

The same, however, could not be said of knee deformities, which are progressive and can preclude walking without aid if not corrected. Successful knee fusion has been reported for a grossly unstable, arthritic, or dislocated knee [24]. However, in patients with multiple comorbidities or chronically infected joints, as well as wheelchair-dependent patients, amputation should also be considered and discussed with the patient, either as a primary procedure or in case of failure of the reconstructive procedures [8, 25–27]. Total knee arthroplasty has been performed in CIP patients and could be a viable option in a non-infected joint [28]. We have not yet performed any joint replacements in CIP patients. However, our purpose in performing guided growth procedures in CIP individuals is to mitigate the need for complex procedures with a high complication rate.

We achieved at least partial correction in all patients. The surgeries were timed to yield a full correction. However, a lower rate of correction was observed in all patients, when compared to patients with idiopathic genu valgum [14]. We observed a 0.28°/month correction rate in the femur, while previous studies have reported a rate of 0.4–0.52°/month [14]. Although anecdotal, we believe that patients with CIP grow at a lower rate, and guided growth procedures should be performed earlier than in non-CIP children.

We observed no major complications related to the surgeries during the study period. Staples had to be removed in one patient due to extrusion [29]. Tension-band plating was performed for four patients, with no hardware-related complications. We recommend using plates for guided growth in CIP patients. Also, none of the patients experienced skin dehiscence or surgical-site infection. We believe that the strict criteria with which we selected candidates for surgery might play a role in this low rate of complications [30]. We continue not to offer surgery to CIP patients with a severe intellectual disability or self-mutilating behavior. One of the patients died 4 years after surgery due to complications of a stabbing incident. Of note, this was the only patient with intellectual disability and behavioral issues in the series.

This study had several limitations. First, this is a small case series with only ten knees in six patients. No causal relationship could be inferred from such a study. However, due to CIP being an exceedingly rare disease, and surgical candidates even more so, this study is significant in introducing a preventive measure for this vulnerable patient population and reporting our clinical experience. Second, the indications for surgery were not standardized. Again, given the heterogeneity of clinical presentation, decisions should be made on a case-by-case basis, while following general guidelines. Third, although we performed surgeries to prevent the progression of knee deformity, we did not have a control group to compare the results with. Furthermore, long-term follow-up is needed to conclude the true success rate of guided growth procedures in CIP patients. It should also be noted that congenital insensitivity to pain (CIP) and hereditary sensory and motor neuropathy (HSAN) were used interchangeably in this paper. Although these terms are not necessarily equivalent, we chose CIP for the title, as orthopedic surgeons might be more familiar with the term, while neurologists might prefer HSAN.

To our knowledge, this is the first study to report the results of guided growth procedures in patients with congenital insensitivity to pain. Our findings suggest that guided growth procedures around the knee have a low complication rate in patients with CIP. The rate of correction was less than half of that predicted for idiopathic genu valgum. Based on our results, we recommend performing guided growth procedures in patients with no intellectual disability or self-mutilating behavior who have a cooperative family to adhere to strict activity limitations and regular follow-ups.

## Data Availability

The datasets used and/or analyzed during the current study are available from the corresponding author on reasonable request.

## Abbreviations

CIP: Congenital insensitivity to pain
HSAN: Hereditary sensory and motor neuropathy
TBP: Tension-band plating
